# A multinational study on the factors influencing university students’ attitudes and usage of ChatGPT

**DOI:** 10.1038/s41598-024-52549-8

**Published:** 2024-01-23

**Authors:** Maram Abdaljaleel, Muna Barakat, Mariam Alsanafi, Nesreen A. Salim, Husam Abazid, Diana Malaeb, Ali Haider Mohammed, Bassam Abdul Rasool Hassan, Abdulrasool M. Wayyes, Sinan Subhi Farhan, Sami El Khatib, Mohamad Rahal, Ali Sahban, Doaa H. Abdelaziz, Noha O. Mansour, Reem AlZayer, Roaa Khalil, Feten Fekih-Romdhane, Rabih Hallit, Souheil Hallit, Malik Sallam

**Affiliations:** 1 Department of Pathology, Microbiology and Forensic Medicine, School of Medicine, The University of Jordan, Amman, 11942 Jordan; 2 Department of Clinical Laboratories and Forensic Medicine, Jordan University Hospital, Amman, 11942 Jordan; 3 Department of Clinical Pharmacy and Therapeutics, Faculty of Pharmacy, Applied Science Private University, Amman, 11931 Jordan; 4 Department of Pharmacy Practice, Faculty of Pharmacy, Kuwait University, Kuwait City, Kuwait; 5 Department of Pharmaceutical Sciences, Public Authority for Applied Education and Training, College of Health Sciences, Safat, Kuwait; 6 Prosthodontic Department, School of Dentistry, The University of Jordan, Amman, 11942 Jordan; 7 Prosthodontic Department, Jordan University Hospital, Amman, 11942 Jordan; 8 College of Pharmacy, Gulf Medical University, P.O. Box 4184, Ajman, United Arab Emirates; 9 School of Pharmacy, Monash University Malaysia, Jalan Lagoon Selatan, 47500 Bandar Sunway, Selangor Darul Ehsan Malaysia; 10 Department of Pharmacy, Al Rafidain University College, Baghdad, 10001 Iraq; 11 Department of Anesthesia, Al Rafidain University College, Baghdad, 10001 Iraq; 12 Department of Biomedical Sciences, School of Arts and Sciences, Lebanese International University, Bekaa, Lebanon; 13 Center for Applied Mathematics and Bioinformatics (CAMB), Gulf University for Science and Technology (GUST), 32093 Hawally, Kuwait; 14 School of Pharmacy, Lebanese International University, Beirut, 961 Lebanon; 15 School of Dentistry, The University of Jordan, Amman, 11942 Jordan; 16 Pharmacy Practice and Clinical Pharmacy Department, Faculty of Pharmacy, Future University in Egypt, Cairo, 11835 Egypt; 17 Department of Clinical Pharmacy, Faculty of Pharmacy, Al-Baha University, Al-Baha, Saudi Arabia; 18 Clinical Pharmacy and Pharmacy Practice Department, Faculty of Pharmacy, Mansoura University, Mansoura, 35516 Egypt; 19 Clinical Pharmacy and Pharmacy Practice Department, Faculty of Pharmacy, Mansoura National University, Dakahlia Governorate, 7723730 Egypt; 20 Clinical Pharmacy Practice, Department of Pharmacy, Mohammed Al-Mana College for Medical Sciences, 34222 Dammam, Saudi Arabia; 21 The Tunisian Center of Early Intervention in Psychosis, Department of Psychiatry “Ibn Omrane”, Razi Hospital, 2010 Manouba, Tunisia; 22 Faculty of Medicine of Tunis, Tunis El Manar University, Tunis, Tunisia; 23 School of Medicine and Medical Sciences, Holy Spirit University of Kaslik, Jounieh, Lebanon; 24 Department of Infectious Disease, Bellevue Medical Center, Mansourieh, Lebanon; 25 Department of Infectious Disease, Notre Dame des Secours, University Hospital Center, Byblos, Lebanon; 26 Research Department, Psychiatric Hospital of the Cross, Jal Eddib, Lebanon

**Keywords:** Human behaviour, Health occupations

## Abstract

Artificial intelligence models, like ChatGPT, have the potential to revolutionize higher education when implemented properly. This study aimed to investigate the factors influencing university students’ attitudes and usage of ChatGPT in Arab countries. The survey instrument “TAME-ChatGPT” was administered to 2240 participants from Iraq, Kuwait, Egypt, Lebanon, and Jordan. Of those, 46.8% heard of ChatGPT, and 52.6% used it before the study. The results indicated that a positive attitude and usage of ChatGPT were determined by factors like ease of use, positive attitude towards technology, social influence, perceived usefulness, behavioral/cognitive influences, low perceived risks, and low anxiety. Confirmatory factor analysis indicated the adequacy of the “TAME-ChatGPT” constructs. Multivariate analysis demonstrated that the attitude towards ChatGPT usage was significantly influenced by country of residence, age, university type, and recent academic performance. This study validated “TAME-ChatGPT” as a useful tool for assessing ChatGPT adoption among university students. The successful integration of ChatGPT in higher education relies on the perceived ease of use, perceived usefulness, positive attitude towards technology, social influence, behavioral/cognitive elements, low anxiety, and minimal perceived risks. Policies for ChatGPT adoption in higher education should be tailored to individual contexts, considering the variations in student attitudes observed in this study.

## Introduction

The rapid advancements in artificial intelligence (AI) and its adoption for teaching and educational purposes could mark a new era of innovation in academia^1–3^. The successful adoption of AI in higher education could pave the way for transformative changes with the potential to reshape the traditional pedagogical methods^4–9^. One of the latest AI-based advancements is ChatGPT—a large language model (LLM) developed by OpenAI—which emerged as a paradigm-shifting innovation for acquisition of information^10–12^.

The LLMs have the potential to revolutionize teaching methodologies in higher education, particularly in fields like health care education^1,13–15^. While AI-based tools could present promising possibilities to reform the teaching and learning processes, these tools are also faced with skepticism and are a subject of ongoing debate due to multiple concerns including ethical issues, factual issues, risk of misinformation spread, copyright issues, among other valid concerns^1,16–19^.

Currently, several challenges are encountered by university students including the issues of rising costs, information overload, the continuous need to acquire and develop new skills, and the limited timeframes for achieving the intended learning outcomes^20–24^. Therefore, novel AI tools like ChatGPT can be valuable to encounter such challenges through increasing efficiency of the learning process with minimal costs and improve the acquisition of new skills by providing a personalized educational experience^1,14,25,26^. Consequently, the need to improve AI literacy among university students appear of paramount importance for competent, ethical, and responsible use of these tools^27,28^.

Multiple studies underlined the significant potential of LLMs such as ChatGPT in higher educational settings^29^. For example, Ray illustrated how ChatGPT can substantially enrich medical education by providing in-depth knowledge on a variety of medical conditions and treatments^30^. In an early systematic review, Sallam concluded that ChatGPT can be advantageous in healthcare education when used under proper academic supervision, especially in refining communication skills^1^. The ease of access of such AI models also presents an opportunity in healthcare education, promoting personalized interaction and thereby encouraging autonomous learning and augmenting group study^1,18^. Additionally, Farrokhniaa et al. conducted a Strengths, Weaknesses, Opportunities, and Threats (SWOT) analysis on ChatGPT identifying its potential in educational settings^31^. Farrokhniaa et al. suggested that ChatGPT can enhance information accessibility, facilitate personalized learning experiences, and reduce teaching burdens, thus streamlining key educational tasks and processes^31^.

On the other hand, valid concerns arise in light of the possible challenges of AI implementation in higher education including the prospect of overreliance on AI assistance which could be associated with compromising the critical thinking and reasoning and decline in the analytical capabilities^1,5,18,32^. This appears as a major issue considering the aim of higher education to enhance cognitive abilities, which could be compromised by excessive dependency on technological tools including the AI-based tools^33–35^.

Additionally, the quality of AI-generated information is another major concern considering the reported factual concerns associated with the use of AI-based tools including ChatGPT^1,19,30^. Moreover, the quality of training datasets used in LLM development could result in the generation of biased content^19,36,37^. Finally, the unequal accessibility to AI-based tools in various societies and regions, could deepen the inequity in education with subsequent psychological and socioecological issues^38–40^.

Several studies and reviews highlighted valid concerns regarding the utility of LLMs, including ChatGPT in higher education^1,18,30,31^. For example, Tlili et al. conducted a thorough investigation into the application of ChatGPT in educational setting^41^. Tlili et al. study involved three analytical approaches: social network analysis of tweets, content analysis of interviews, and a detailed examination of user experiences, particularly focusing on ChatGPT's early adopters in educational contexts^41^. While recognizing ChatGPT’s efficacy in education, Tlili et al. highlighted that ChatGPT implementation in education necessitates vigilance with the formulation of more robust usage guidelines^41^. Furthermore, Farrokhniaa et al. in their SWOT analysis, identified several potential threats posed by ChatGPT to the educational sector, including the challenges to understand context, risks to academic integrity, potential reinforcement of educational biases, facilitation of plagiarism, and a possible decline in advanced cognitive skills^31^.

The successful integration and acceptance of innovative tools such as ChatGPT within educational settings can be influenced by a variety of factors among both the students and instructors^42–44^. For example, an important factor precluding the use of ChatGPT can be the perception of possible risks (e.g., security risks, privacy concerns, unreliability of information, risk of accusation of plagiarism and violation of academic policies)^1,14,45,46^. Thus, the perceived risk of ChatGPT use can be a decisive factor for its adoption in the teaching and learning processes^1,18,47,48^. Another important factor is the perceived ease of use, which is an important factor driving the acceptance of this novel tool in education^49^.

Additionally, the perceived usefulness can be a significant driving factor in the adoption of ChatGPT in the learning process through facilitating academic activities and assignments while saving time^50–52^. Furthermore, a complex array of cognitive and behavioral determinants as well as the perceived enjoyment, social influence and attitude towards technology in general can be viewed as important determinants for the acceptance of a novel technology such as ChatGPT^53–55^.

To unravel the multifaceted aspects driving the adoption of ChatGPT among university students for educational purposes, a study validated a survey instrument based on the technology acceptance model (TAM)^51,56^. This instrument, termed “TAME-ChatGPT” (Technology Acceptance Model Edited to Assess ChatGPT Adoption) dissected a wide range of factors that could influence university students’ attitudes and behaviors towards ChatGPT and its usage^51^.

Therefore, the primary objective of the current study was to analyze the extent and determinants of ChatGPT usage among university students in Arab-speaking countries. The study aimed to provide deeper insights that can inform educators, policymakers, and academic institutions on the possibilities and concerns regarding ChatGPT integration within the academia. The study objectives included confirming the validity of TAME-ChatGPT survey instrument conceived to improve the understanding of the complex factors influencing the adoption of ChatGPT in educational settings from the students’ perspective.

The current study was distinctive through the methodical approach employing the “TAME-ChatGPT” instrument^51^. This survey instrument was specifically designed to evaluate the attitudes towards ChatGPT and its adoption among university students^51^, facilitating a detailed comprehension of the various factors that could shape university students’ perceptions and interactions with ChatGPT.

Additionally, the focus on university students in Arab-speaking countries aimed to shed light on the cultural and linguistic factors that could influence technology adoption in educational settings. Thus, the study objectives extend beyond merely validating the “TAME-ChatGPT” instrument, since it aimed to provide valuable insights for educators, policy makers, and academic institutions regarding the implementation of ChatGPT in academic contexts, with a special focus on a region that might be underrepresented in such a research inquiry.

## Results

### Characteristics of the study sample

The final study sample comprised a total of 2240 participants who completed the survey representing five countries (Egypt, Iraq, Jordan, Kuwait, and Lebanon), with a mean age of 22.25 ± 4.58 years and 72.1% females (*n* = 1615). Moreover 46.8% have heard about ChatGPT, of which 52.6% indicated using ChatGPT before participation in the study. Other characteristics of the sample can be found in (Table 1).

**Table 1:** Sociodemographic and other characteristics of the participants (n = 2240).

Characteristic	Number (%)
Country	
Egypt	417 (18.6%)
Iraq	736 (32.9%)
Jordan	242 (10.8%)
Kuwait	582 (26.0%)
Lebanon	263 (11.7%)
Sex	
Male	625 (27.9%)
Female	1615 (72.1%)
University	
Public	983 (43.9%)
Private	1257 (56.1%)
Self-reported latest GPA	
Excellent	537 (24.0%)
Very good	765 (34.2%)
Good	759 (33.9%)
Satisfactory	138 (6.2%)
Unsatisfactory	31 (1.4%)
Have heard of ChatGPT (yes)	1048 (46.8%)
Have used ChatGPT (yes)	551 (52.6%) *

	Mean ± SD
Age (years)	22.25 ± 4.58
Perceived usefulness*	23.30 ± 4.65
Behavior*	9.77 ± 3.03
Perceived risk of use*	7.56 ± 2.87
Perceived ease of use*	8.98 ± 1.30
Anxiety	6.97 ± 3.04
Technology social influence	19.72 ± 3.74
Perceived risk	12.43 ± 4.41

*Among those who have heard of ChatGPT before the study; *GPA* grade point average, *SD* standard deviation.

### General description of the TAME-ChatGPT scores in the study sample

**Figure 1:**
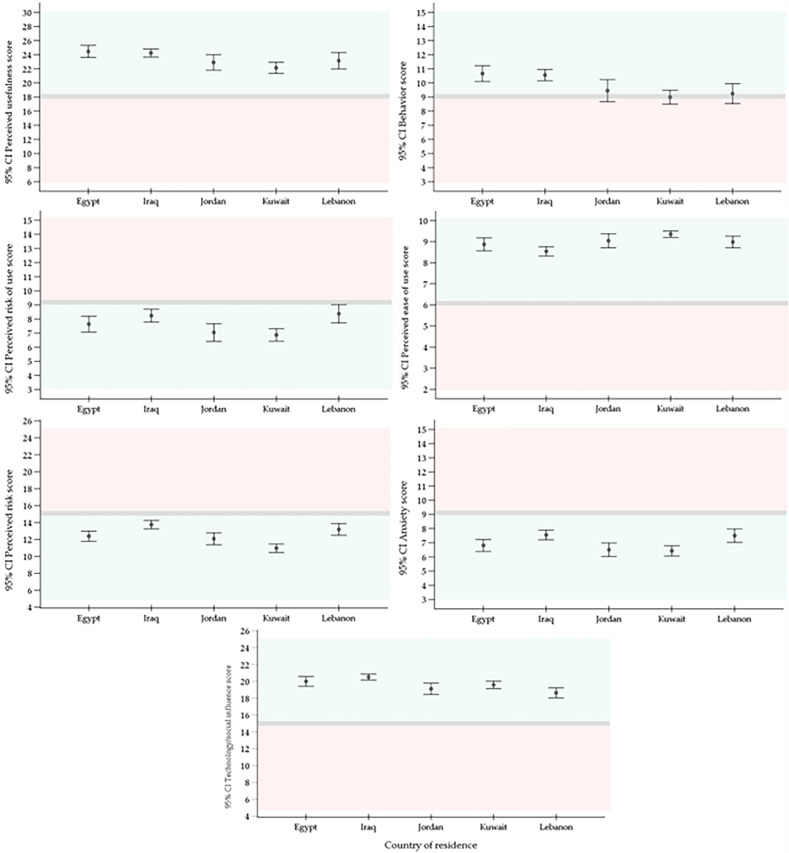
Descriptive analyses of the key TAME-ChatGPT constructs’ scores stratified by country of residence for the participants. *CI*: confidence interval of the mean. Positive attitude is highlighted in light green, negative attitude in light red, and neutral attitude in grey.

Descriptive analyses of the key TAME-ChatGPT constructs’ scores revealed a generally positive attitude towards ChatGPT and its use in the study sample, as reflected in Table 2 and Fig. 1.

**Table 2:** Descriptive statistics of the TAME-ChatGPT constructs in the study sample.

Construct	Perceived usefulness	Behavior score	Perceived risk of use	Perceived ease of use	General perceived risk	Anxiety	Technology/social influence
Number	551	551	551	551	1048	1048	1048
Mean ± SD	23.3 ± 4.6	9.8 ± 3.0	7.6 ± 2.9	9.0 ± 1.3	12.4 ± 4.4	7.0 ± 3.0	19.7 ± 3.7
Median	24	10	7	10	12	6	20
Minimum	6	3	3	2	5	3	5
Maximum	30	15	15	10	25	15	25
IQR	21–27	8–12	5–9	8–10	9–15	5–9	18–23
Attitude	Agreement	Positive influence	Low perceived risk	Agreement	Low perceived risk	Low anxiety	Positive influence

*SD* standard deviation; *IQR* interquartile range.

### Confirmatory factor analysis

**Figure 2:**
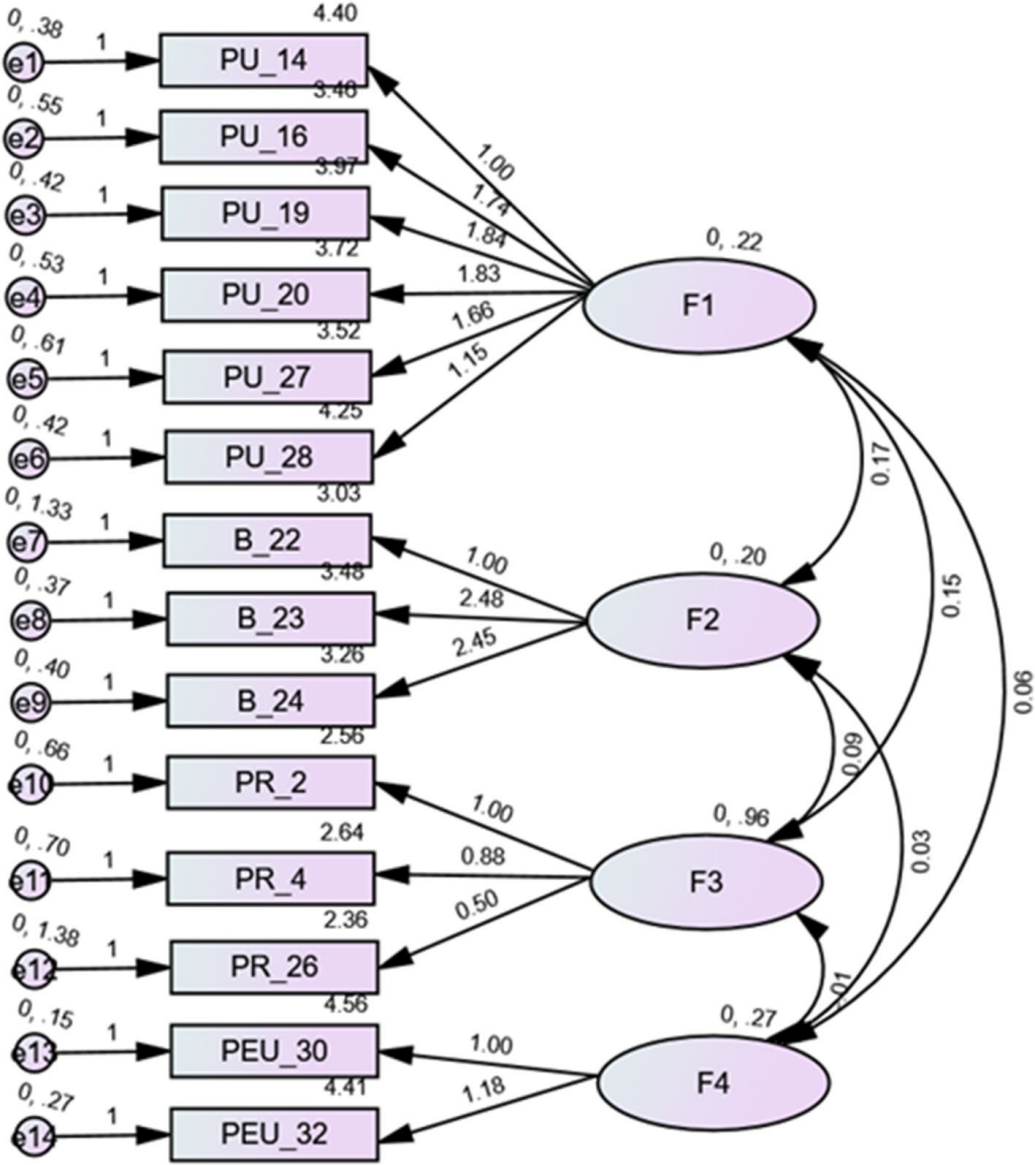
Items of the ChatGPT usage scale and standardized estimates of factor loadings from the confirmatory factor analysis (CFA) in participants who have used ChatGPT. *F1*: Perceived usefulness (PU), *F2*: Behavioral/cognitive factors (B), *F3*: Perceived risk of use (PR), *F4*: Perceived ease of use (PEU).

**Figure 3:**
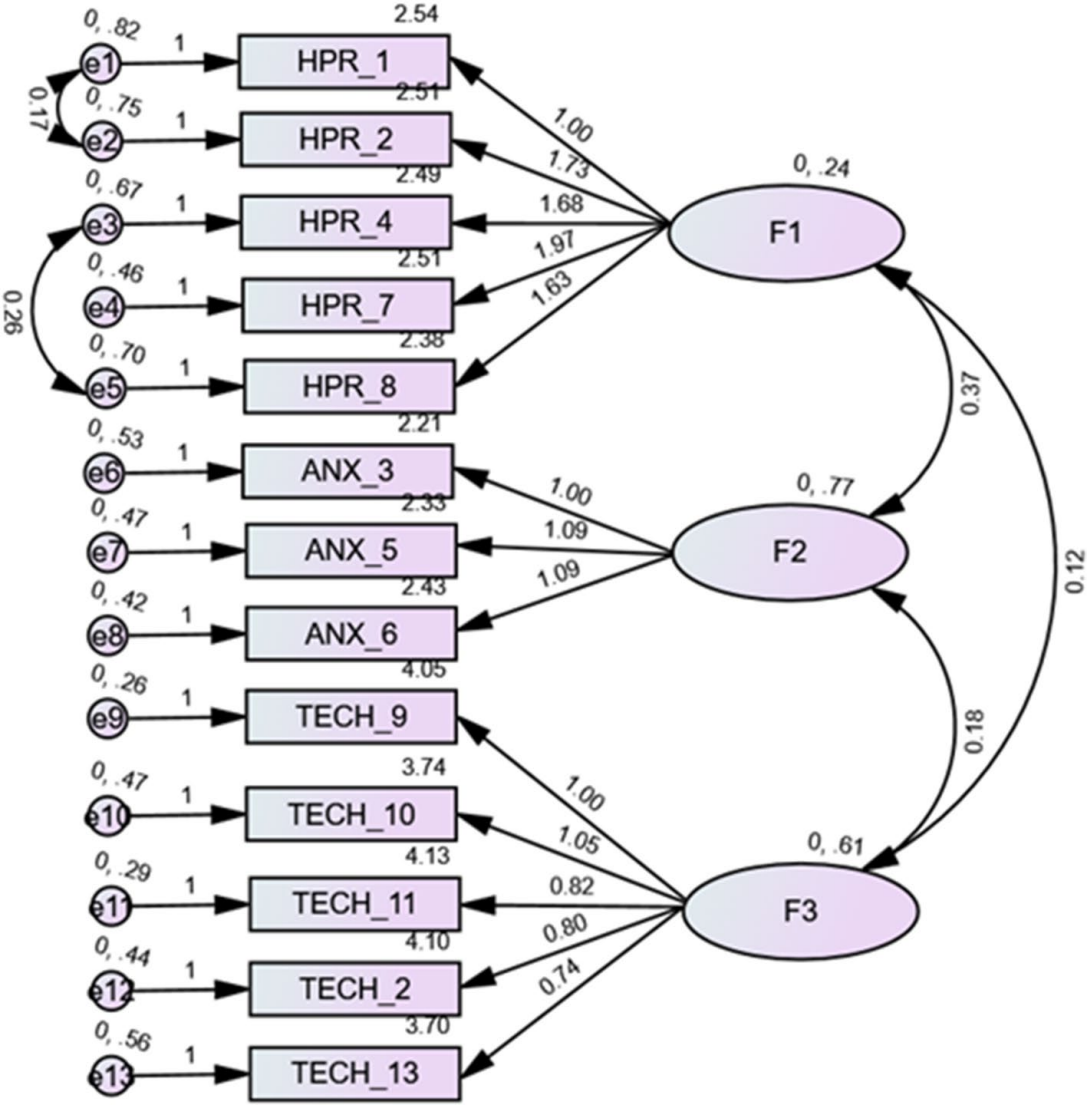
Items of the ChatGPT attitude scale and standardized estimates of factor loadings from the confirmatory factor analysis (CFA) in participants who have heard about ChatGPT. *F1*: Perceived risk in general (HPR), *F2*: Anxiety (ANX), *F3*: Attitude to technology/social influence (TECH).

The CFA results of the TAME-ChatGPT usage scale was conducted on those who have used ChatGPT (*n* = 551). The fit indices were adequate as follows: χ^2^/df = 300.20/71 = 4.23, *p* < 0.001, RMSEA = 0.077 (90% CI 0.068–0.086), SRMR = 0.050, CFI = 0.923, and TLI = 0.901. The standardized estimates of factor loadings are shown in (Fig. 2). The CFA results of the attitude scale was conducted on those who have used ChatGPT (*n* = 1048). The fit indices were adequate as follows: χ^2^/df = 436.67/62 = 7.04, *p* < 0.001, RMSEA = 0.076 (90% CI 0.069–0.083), SRMR = 0.038, CFI = 0.942, and TLI = 0.927. When adding correlations between the items 1–2 and 4–8, the fit indices improved as follows: χ^2^/df = 288.28/60 = 4.81, *p* < 0.001, RMSEA = 0.060 (90% CI 0.053–0.067), SRMR = 0.032, CFI = 0.965, and TLI = 0.954. The standardized estimates of factor loadings are shown in (Fig. 3).

### Bivariate analysis of factors associated with ChatGPT usage

The results of the bivariate analysis are summarized in Table 3, which showed a statistically significant differences in ChatGPT usage scale based on country of residence, type of university, and self-reported GPA. A higher mean ChatGPT usage total score was found in Egypt compared to the other countries, in students from private universities and in those who have satisfactory GPA. Moreover, older age was significantly associated with lower ChatGPT usage scores (*r* = − 0.18; *p* < 0.001).

**Table 3:** Bivariate analysis of factors associated with ChatGPT usage (n = 551).

	Mean ± SD	*t/F*	*df/df1, df2*	*p*
Country		7.202	4, 546	**< 0.001**
Egypt	51.65 ± 7.01			
Iraq	51.58 ± 6.54			
Jordan	48.46 ± 8.40			
Kuwait	47.36 ± 9.59			
Lebanon	49.75 ± 8.55			
Sex		1.263	549	0.207
Male	50.15 ± 7.87			
Female	49.24 ± 8.69			
University		− 3.878	549	**< 0.001**
Public	48.29 ± 8.93			
Private	51.03 ± 7.48			
Latest GPA		3.312	4, 545	**0.011**
Excellent	47.77 ± 9.38			
Very good	49.95 ± 8.11			
Good	50.90 ± 6.91			
Satisfactory	51.50 ± 9.15			
Unsatisfactory	48.71 ± 12.32			

Numbers in bold indicate significant *p* values. *GPA* self-reported latest point grade average.

### Multivariable analysis

Being from Iraq (Beta = − 2.91), Jordan (Beta = − 4.77), Kuwait (Beta = − 5.00) and Lebanon (Beta = − 4.58) compared to Egypt and older age (Beta = − 0.11) were significantly associated with lower ChatGPT usage total scores. Moreover, having a very good (Beta = 1.73) and good (Beta = 2.47) GPA compared to excellent was significantly associated with higher ChatGPT usage total scores (Table 4).

**Table 4:** Multivariable linear regression taking the ChatGPT usage score as the dependent variable (R^2^ = 0.117) (n = 551).

	Unstandardized Beta	Standardized Beta	*p*	95% CI	VIF
Sex (females vs males*)	− 0.81	− 0.06	0.108	− 1.80; 0.18	1.068
Iraq vs Egypt*	− 2.91	− 0.15	**0.002**	− 4.73; − 1.08	2.121
Jordan vs Egypt*	− 4.77	− 0.18	**< 0.001**	− 6.68; − 2.85	1.247
Kuwait vs Egypt*	− 5.00	− 0.29	**< 0.001**	− 6.47; − 3.54	1.617
Lebanon vs Egypt*	− 4.58	− 0.18	**< 0.001**	− 6.70; − 2.46	1.578
University (private vs public*)	0.83	0.06	0.223	− 0.50; 2.16	1.914
GPA (very good vs excellent*)	1.73	0.12	**0.009**	0.44; 3.02	1.727
GPA (good vs excellent*)	2.47	0.16	**0.001**	1.07; 3.87	1.821
GPA (satisfactory vs excellent*)	2.49	0.08	0.033	0.21; 4.78	1.235
GPA (unsatisfactory vs excellent*)	− 0.72	− 0.01	0.788	− 5.96; 4.52	1.039
Age	− 0.11	− 0.11	**0.012**	− 0.19; − 0.02	1.570

*GPA* self-reported latest point grade average, *VIF* variance inflation factor, *CI* confidence interval.

Significant *p* values are highlighted in bold.

## Discussion

In this study, a slightly less than a quarter of the participating students indicated the use of ChatGPT highlighting the widespread adoption of this LLM-based tool, previously recognized as the most rapidly expanding consumer application in history^57–59^. This versatility of ChatGPT use or the intention to use it as an aid in university assignments was illustrated recently in a large multinational study by Ibrahim et al.^60^. This multinational study that was conducted among academics and students in Brazil, India, Japan, UK, and USA, regarding their perspectives on ChatGPT, indicated that a majority of students intend to use ChatGPT for assignment support and anticipate that their peers would endorse its usage, implying a potential shift towards ChatGPT use becoming a standard practice among university students^60^. Recently, Strzelecki meticulously delineated the factors behind adoption of ChatGPT among Polish state university students^61^. The study revealed that habit had the greatest impact on behavioral intention to adopt ChatGPT, followed by performance expectancy and hedonic motivation^61^. For the behavior of use, the most significant factors were the behavioral intention, habit, and facilitating conditions^61^. Another relevant and rigorous study among university students in the UAE by Farhi et al. showed that ChatGPT use significantly affected the students’ views, concerns, and perceived ethics towards such a revolutionary technology^62^.

Several previous studies indicated the potential utility of ChatGPT as a prime example among other LLMs in higher education^1,18,26,63–65^. For example, Montenegro-Rueda et al. highlighted the potential for ChatGPT to facilitate the interaction between students and teachers besides being a motivational tool in the learning process^26^. In a relevant editorial, Meyer et al. pointed out that the main issue regarding LLMs’ use in academia would be approach by which students employ these models^63^. Meyer et al. emphasized the importance of students’ engagement as prompt creators and fact checkers in an educational framework, rather than simply relying on AI-produced material^63^. Multiple recent studies highlighted the need to revise the current assessment methods in higher education in light of the high performance of LLMs in various exams^66–69^.

Based on the prospects of ChatGPT in higher education, a previous study explored the validity of a survey instrument to assess the factors influencing the adoption of this novel tool among university students in health schools in Jordan^51^. The current study confirmed the validity of this survey instrument termed “TAME-ChatGPT” as a valuable tool to elucidate the determinants of ChatGPT use and attitude towards this novel AI-based conversational model.

In this study, the major findings illustrated that the adoption of ChatGPT among university students is influenced by both socio-demographic variables and various TAM constructs as modeled in “TAME-ChatGPT”. Additionally, the study findings revealed that ChatGPT was perceived to have both positive and negative aspects among the participating students reflecting the ongoing debate regarding ChatGPT^1,14^. This appears conceivable in light of the current evidence showing that the use of AI-based tools for educational purposes were perceived as a double-edged sword^1,5,14,70^. On one hand, these tools can be valuable in delivering timely, efficient, and personalized support to a broad student population promoting equity in education^71–74^. On the other hand, valid concerns should be emphasized including the possible generation of inaccurate and biased educational content among other ethical concerns^1,5,75,76^. Additionally, Safranek et al. highlighted the current limitations of ChatGPT in medical education including the inability to provide comprehensive contextual information along with its lack of intuitive patient assessment capabilities which are essential skills acquired during medical education^75^. An early systematic review by Sallam emphasized the relatively below bar performance of ChatGPT in some topics hindering its current utility in healthcare education^1^. Similarly, multiple later studies confirmed this concern of generating inaccurate content in specific topics (e.g., Radiology, Microbiology)^77–79^.

To successfully exploit the potential of ChatGPT in the learning and teaching processes, the current study revealed the following relevant factors: First, one of the most promising features of ChatGPT is its ease of use, which was reflected by general agreement of a majority of the participants students in this study. The ease of ChatGPT use is a notable feature of this tool promoting its widespread accessibility and usability^58^. As previously illustrated in various studies, ChatGPT responds to queries in various languages, with notable capabilities facilitating the generation of coherent responses^11,30,80–82^. A study among university students in Jordan by Ajlouni et al. showed that a majority of participants (73%) agreed on the potential of ChatGPT in facilitating the learning process^83^. As a “smart” user-friendly tool, ChatGPT has been shown to be suitable for a wide range of applications, including answering questions, text generation, and aiding in writing of various tasks^30,84,85^. Thus, it is conceivable that this particular construct showed a high score among the study sample in various settings.

Based on the findings of the current study, the incorporation of ChatGPT in the learning process among university students can benefit from the ease-of-use feature which was identified as a major factor driving ChatGPT use in the study sample. This finding is in line with results of previous studies which showed that effort expectancy was an important determinant of adoption of novel educational technologies including ChatGPT^64,86,87^.

The user-friendly nature of ChatGPT facilitate its immediate accessibility to students of varying backgrounds^30^. Through providing an immediate source for clarifying complex concepts, ChatGPT can reduce barriers to learning in higher education^25^. The ease-of-use can also offer a personalized learning experience that addresses individual student needs and preferences. Taking into consideration the current study setting in Arab-speaking countries, and based on English language prominence in higher education, ChatGPT can be a valuable tool assisting non-native English speakers to improve the learning process, thereby promoting inclusivity and equity in higher education^76,88,89^. Furthermore, the prompt ability of ChatGPT in information retrieval and content generation can allow university students to allocate more time to understand complex educational materials leading to more effective achievement of the intended learning outcomes^1,25,90^.

Second, another major determinant of ChatGPT use among the participating students in this study was the perceived usefulness of this novel tool via providing accuracy and speed. Numerous previous studies highlighted that the perceived usefulness of a novel technology is a key factor influencing the intention of users to adopt such a technology^50,91,92^.

The study findings highlighted the versatile advantages of ChatGPT in supporting academic tasks among university students. This was reflected by generally high agreement of the participants on the “perceived usefulness” construct items, highlighting that ChatGPT could enhance efficiency in university assignments and duties, aligning with students’ beliefs regarding usefulness of ChatGPT for educational purposes^14,60^.

Third, in this study, the positive attitude towards technology as well as the social influence were found as major factors driving the adoption of ChatGPT among the university students. A majority of the sample scored high on the “attitude towards technology/social influence” construct. The responses from participants in this study emphasized the key role of readiness to accept novel technological tools in achieving academic success. This result is conceivable considering that the inclination to embrace novel technological tools, as well as the influence of peers, collectively emerge as key determinants contributing to a successful adoption of new technologies within an educational context^93–95^.

Fourth, among the other factors identified as important determinants for ChatGPT adoption among university students in this study were the behavioral/cognitive factors. Certain behavioral and cognitive factors, such as habits, beliefs, and thought processes, are expected to play a significant role in shaping the attitude towards a novel technology such as ChatGPT^96–98^. Therefore, it is expected that participants who reported prior experience with tools similar to ChatGPT could be more comfortable and familiar with such a novel technology, rendering those students more likely to adopt ChatGPT for educational purposes. Moreover, the spontaneous use of ChatGPT to retrieve information for academic assignments suggests an intrinsic inclination to rely on the tool, indicating a cognitive readiness to integrate it among university students as indicated by the recent multinational study by Ibrahim et al., which showed that the majority of students (> 90%) intended to use ChatGPT as an aiding tool in their assignments in the coming semester^60^.

Fifth, the generally low perceived risks and low anxiety levels among the participating university students in this study suggest a readiness to adopt ChatGPT, in spite of the recognized concerns and known risks associated with this novel AI-based technology^99,100^. These concerns that were shown previously included possible unreliability of the generated content, risk of plagiarism, security concerns, risk of violating the academic policies, and privacy issues when using ChatGPT^5,14,18^. The finding of low perceived risks in the study sample suggest that the aforementioned concerns were not strongly perceived among students in the sample and indicate the readiness to embrace ChatGPT in the learning process despite the appreciated concerns.

Furthermore, the generally low “anxiety” scores, including the fear of declining the critical thinking skills, over-dependence on technology, and diminished originality in assignments, suggest that the participating students were not anxious about these potential drawbacks. Instead, the study findings suggest that university students could view ChatGPT as a valuable tool in education with low perceived anxiety regarding possible breaches of academic integrity or issues in the development of their skills.

In multivariate analysis, the usage of ChatGPT was higher among the students based in Egypt possibly reflecting heightened perceived ease of ChatGPT use, usefulness, familiarity with technological advancements, and low perceived risks for this AL model. This association could point to distinct cultural and educational aspects prevalent across different countries.

Finally, if ChatGPT among other relevant LLM are to be implemented as a tool for educational purposes, the study findings suggest that the university policies should be tailored in various settings and based on factors such as age and academic performance as reflected by GPA. Different age groups of university students may have varying needs, preferences, and different levels of familiarity with technological advancements. Tailoring policies to accommodate these generational disparities can enhance the overall student experience and acceptance of ChatGPT. Additionally, students with diverse academic achievements may have distinct requirements for utilizing ChatGPT effectively. Customizing policies that address these variations can promote equitable academic achievements and ensure that the tool aligns with students’ academic goals^14^.

Limitations of the study requires careful considerations upon attempting to interpret the findings. These limitations included the approach of sampling which was convenience-based. Such an approach is limited by possible selection bias with subsequent lack of generalizability; however, the selection of this sampling approach was based on cost issues, efficiency, and being simple to implement^101^. Other limitations included the cross-sectional design, limiting the ability to establish causality or to explore the temporal changes in attitudes and usage patterns of ChatGPT. Additionally, the possible response bias should be considered in light of the possibility of perceived social desirability, in light of the controversy surrounding ChatGPT use in academia. Moreover, the current study relied on self-reported data by the participants, which can be subject to biases including over- or under-estimation of participants’ usage and attitudes towards ChatGPT. Furthermore, the varying levels of ChatGPT experience among the participants represent a critical factor that was not evaluated in the study. Such variability levels could have significantly influenced the participants’ attitudes towards ChatGPT, an aspect that is worth considering in future studies for a comprehensive understanding of attitudes towards ChatGPT.

The successful adoption of ChatGPT among university students is expected to be related to multifaceted factors as intricately inferred through the validated “TAME-ChatGPT” instrument. These factors include the highly perceived ease of use, perceived usefulness, positive attitude towards technology in general together with the effect of social influence, and the low anxiety and the low perceived risks. Understanding the dynamic interplay of these factors is important for higher education institutions, educators, policymakers, and other stakeholders if they attempt the integration of AI technologies into educational practices. These TAM-based factors together with demographic factors could collectively influence the students’ attitudes towards ChatGPT, rendering them more likely to view it positively and use it beneficially to achieve the intended learning outcomes in academic settings.

## Methods

### Study design

The current study employed a cross-sectional design with an electronic distribution of a previously validated survey instrument^51^. The “TAME-ChatGPT” instrument has been shown as a reliable, valid, and practical tool to assess university students’ attitudes towards ChatGPT^51^. Specifically, the TAME-ChatGPT tool helps to unravel the role of factors such as risk perceptions, perceived usefulness, ease of use, attitudes towards technology, and behavioral aspects in the adoption of ChatGPT as an educational tool among students^51^.

The sample was collected using a non-probability sampling (convenience-based approach). The survey was hosted in Google Forms and distributed by the authors from multiple Arab countries (Egypt, Iraq, Jordan, Kuwait, Lebanon, Saudi Arabia, and Tunisia). The cut-off for inclusion of participants in the sample per country was set at a minimum of 125 valid responses based on the number of items in the original TAME-ChatGPT scale (25 items)^51^. A minimum sample size of 125 participants (5 participants per item) was considered essential to maintain the statistical rigor and ensure the robustness of the confirmatory factor analysis (CFA) results, which would allow an accurate estimation of model parameters and factor loadings^102,103^.

The self-administered questionnaire was provided concurrently in Arabic and English languages. The study participants were conveniently recruited through the authors’ network in Arab countries (a majority of which were either instructors or students in Arab universities). To reach the potential participants, the survey link was disseminated via social media and instant messaging services (Facebook, Twitter, LinkedIn, WhatsApp, and Messenger) directed to university students in Arab countries. The survey link was accessible from 24 April 2023, until 15 August 2023, and participation was entirely voluntary, without any incentives for participation.

The inclusion criteria, as explicitly outlined at the beginning of the questionnaire prior to seeking informed consent, clearly stated that participants must meet the following conditions: (1) an age of 18 years or older, (2) to be currently enrolled in a university in one of Arab countries (Appendix S1).

### Questionnaire structure

Following the introduction highlighting the aim of the study, a mandatory informed consent item was introduced “Do you agree to participate in this study?” with “yes” as answer being required to move into the next section of the survey, while the answer of “no” resulting in closure of the survey.

The next section assessed the socio-demographic features of the participants. The following variables were assessed: (1) age as a scale variable; (2) sex (male vs. female); (3) current country of residence (Algeria, Bahrain, Djibouti, Egypt, Iraq, Jordan, Kuwait, Lebanon, Libya, Mauritania, Morocco, Oman, Palestine, Qatar, Saudi Arabia, Somalia, Sudan, Syria, Tunisia, the United Arab Emirates (UAE), and Yemen); (4) ethnicity (Arab vs. non-Arab); (5) School/College/Faculty (health vs. scientific vs. humanities); (6) University (public vs. private); (7) current educational level (bachelor (BSc) vs. masters (MSc) vs. doctorate (PhD)); (8) The latest grade point average (GPA) (excellent, very good, good, satisfactory, and unsatisfactory).

This was followed by two questions: have you heard of ChatGPT before the study? (Yes vs. No) with an answer of “No” resulting in submission of the response and closure of the survey. An answer of “Yes” resulted in movement to the next question “Have you used ChatGPT before the study?” (Yes vs. No). An answer of “No” resulted in moving into the attitude scale questions (13 items), while the answer of “yes” resulted in moving into the attitude and usage scale questions altogether (25 items). The items comprising the constructs of TAME-ChatGPT is shown in (Appendix S1). Each scale item was assessed using a 5-point Likert scale, where “agree” corresponded to a score of 5, “somewhat agree” to 4, “neutral/no opinion” to 3, “somewhat disagree” to 2, and “disagree” to 1. Conversely, the scoring was reversed for the items indicating a negative attitude (Appendix S1).

### Ethics statement

The current study was approved by the Institutional Review Board at the Faculty of Pharmacy—Applied Science Private University (approval number: 2023-PHA-21). In the introductory section of the survey, the following issues were clearly stated: (1) assurance of the confidentiality and anonymity of the responses; (2) confirmation of the participant status as current university students in an Arab country; (3) confirmation of voluntary participation in the survey. This was followed by the mandatory informed consent question “Do you agree to participate in this study?” which was necessary for completion of the survey.

### Statistical and data analysis

The statistical analysis was conducted using IBM SPSS Statistics for Windows, Version 26.0. Armonk, NY: IBM Corp. AMOS was used to conduct the CFA and to analyze the fitness of models.

Measures of central tendency (mean, median) and dispersion (SD, IQR) were used for descriptive statistics. Seven constructs were evaluated as scale variables for those who heard of ChatGPT as follows (the first four constructs were assessed only among those who used ChatGPT):

(1) Perceived usefulness comprising six items with a maximum score of 30 indicating agreement that ChatGPT is useful, a score of 18 indicating neutral attitude to the usefulness of ChatGPT and a score of 6 indicating disagreement that ChatGPT is useful; (2) Behavioral/cognitive factors comprising three items with a maximum score of 15 indicating higher role of these factors as determinants of ChatGPT use, a score of 9 indicating that these factors neither strongly influence nor discourage the use of ChatGPT and a score of 3 indicating minimal impact of these factors as determinants of ChatGPT use; (3) Perceived risk of use comprising three items, which were reverse coded with a maximum score of 15 indicating high perceived risks in relation to ChatGPT use, a score of 9 indicating neutral attitude towards the perceived risks of ChatGPT use and a score of 3 indicating low perceived risks in relation to ChatGPT use; (4) Perceived ease of use comprising two items, with a maximum score of 10 indicating agreement that ChatGPT is easy to use, a score of 6 indicating a neutral attitude towards the ease of ChatGPT use of ChatGPT and a score of 2 indicating disagreement that ChatGPT is easy to use; (5) General perceived risks, comprising five items which were reverse coded with a maximum score of 25 indicating high perceived risks in relation to ChatGPT in general, a score of 15 indicating neutral attitude towards the perceived risks of ChatGPT and a score of 5 indicating low perceived risks in relation to ChatGPT in general; (6) Anxiety comprising three items, which were reverse coded with a maximum score of 15 indicating high anxiety in relation to ChatGPT as a technological tool, a score of 9 indicating neutral attitude and a score of 3 indicating low anxiety in relation to ChatGPT; and (7) Attitude to technology and social influence comprising five items with a maximum score of 25 indicating positive attitude towards technology and higher role of the social influence, a score of 15 indicating neutral attitude a score of 5 indicating negative attitude towards technology and lower role of the social influence.

The CFA was employed to assess the structural validity of the TAME-ChatGPT constructs. Specifically, CFA for the usage sub-scales was conducted among ChatGPT users (n = 551), while CFA for the attitude sub-scales was conducted among those who heard of ChatGPT (n = 1048). The following model fit indices were employed: χ^2^/degree of freedom (df), root mean square error of approximation (RMSEA), standardized root mean square residual (SRMR), comparative fit index (CFI), and Tucker-Lewis index (TLI). Standardized factor loadings for each scale item were also determined. Multivariable regression analysis was performed to investigate the possible factors influencing ChatGPT usage scores. The variables considered in this analysis included participants’ country of origin, age, and GPA.

### Institutional review board statement

The study was conducted in accordance with the Declaration of Helsinki and approved by the Institutional Review Board at the Faculty of Pharmacy—Applied Science Private University (approval number: 2023-PHA-21, date of approval: May 2023).

### Informed consent

Informed consent was obtained from all subjects involved in the study through a mandatory item in the survey necessary for successful completion and submission of the response.

### Supplementary Information

Supplementary Information.

## Data Availability

The data presented in this study are available on request from the corresponding author (M.S.).
