# Author Correction: A multinational study on the factors influencing university students’ attitudes and usage of ChatGPT

**DOI:** 10.1038/s41598-024-59011-9

**Published:** 2024-04-09

**Authors:** Maram Abdaljaleel, Muna Barakat, Mariam Alsanafi, Nesreen A. Salim, Husam Abazid, Diana Malaeb, Ali Haider Mohammed, Bassam Abdul Rasool Hassan, Abdulrasool M. Wayyes, Sinan Subhi Farhan, Sami El Khatib, Mohamad Rahal, Ali Sahban, Doaa H. Abdelaziz, Noha O. Mansour, Reem AlZayer, Roaa Khalil, Feten Fekih-Romdhane, Rabih Hallit, Souheil Hallit, Malik Sallam

**Affiliations:** 1 Department of Pathology, Microbiology and Forensic Medicine, School of Medicine, The University of Jordan, Amman, 11942 Jordan; 2 Department of Clinical Laboratories and Forensic Medicine, Jordan University Hospital, Amman, 11942 Jordan; 3 Department of Clinical Pharmacy and Therapeutics, Faculty of Pharmacy, Applied Science Private University, Amman, 11931 Jordan; 4 Department of Pharmacy Practice, Faculty of Pharmacy, Kuwait University, Kuwait City, Kuwait; 5 Department of Pharmaceutical Sciences, Public Authority for Applied Education and Training, College of Health Sciences, Safat, Kuwait; 6 Prosthodontic Department, School of Dentistry, The University of Jordan, Amman, 11942 Jordan; 7 Prosthodontic Department, Jordan University Hospital, Amman, 11942 Jordan; 8 College of Pharmacy, Gulf Medical University, P.O. Box 4184, Ajman, United Arab Emirates; 9 School of Pharmacy, Monash University Malaysia, Jalan Lagoon Selatan, 47500 Bandar Sunway, Selangor Darul Ehsan Malaysia; 10 Department of Pharmacy, Al Rafidain University College, Baghdad, 10001 Iraq; 11 Department of Anesthesia, Al Rafidain University College, Baghdad, 10001 Iraq; 12 Department of Biomedical Sciences, School of Arts and Sciences, Lebanese International University, Bekaa, Lebanon; 13 Center for Applied Mathematics and Bioinformatics (CAMB), Gulf University for Science and Technology (GUST), 32093 Hawally, Kuwait; 14 School of Pharmacy, Lebanese International University, Beirut, 961 Lebanon; 15 School of Dentistry, The University of Jordan, Amman, 11942 Jordan; 16 Pharmacy Practice and Clinical Pharmacy Department, Faculty of Pharmacy, Future University in Egypt, Cairo, 11835 Egypt; 17 Department of Clinical Pharmacy, Faculty of Pharmacy, Al-Baha University, Al-Baha, Saudi Arabia; 18 Clinical Pharmacy and Pharmacy Practice Department, Faculty of Pharmacy, Mansoura University, Mansoura, 35516 Egypt; 19 Clinical Pharmacy and Pharmacy Practice Department, Faculty of Pharmacy, Mansoura National University, Dakahlia Governorate, 7723730 Egypt; 20 Clinical Pharmacy Practice, Department of Pharmacy, Mohammed Al-Mana College for Medical Sciences, 34222 Dammam, Saudi Arabia; 21 The Tunisian Center of Early Intervention in Psychosis, Department of Psychiatry “Ibn Omrane”, Razi Hospital, 2010 Manouba, Tunisia; 22 Faculty of Medicine of Tunis, Tunis El Manar University, Tunis, Tunisia; 23 School of Medicine and Medical Sciences, Holy Spirit University of Kaslik, Jounieh, Lebanon; 24 Department of Infectious Disease, Bellevue Medical Center, Mansourieh, Lebanon; 25 Department of Infectious Disease, Notre Dame des Secours, University Hospital Center, Byblos, Lebanon; 26 Research Department, Psychiatric Hospital of the Cross, Jal Eddib, Lebanon

Correction to: *Scientific Reports* 10.1038/s41598-024-52549-8, published online 23 January 2024

The original version of this Article contained an error in Affiliation 17, which was incorrectly given as ‘Department of Clinical Pharmacy, The National Hepatology and Tropical Medicine Research Institute, Cairo, 11835, Egypt’. The correct affiliation is listed below:

Department of Clinical Pharmacy, Faculty of Pharmacy, Al-Baha University, Al-Baha, Saudi Arabia.

The original Article has been corrected.

