# Supplementary material for: A multinational study on the factors influencing university students’ attitudes and usage of ChatGPT
Source: Sci Rep. 2024 Jan 23;14:1983. doi: 10.1038/s41598-024-52549-8 (PMC10806219; doi:10.1038/s41598-024-52549-8)
Supplement: Supplementary file 1 — Supplementary Information. [file 41598_2024_52549_MOESM1_ESM.docx]

**Appendix S1.** **Consent form and TAME-ChatGPT scale**

Thank you for your interest in participating in this study...

The researchers in this study aim to investigate the attitude of university students who are currently studying in universities in Arab countries towards the ChatGPT, which is based on artificial intelligence...

Time required to complete the survey: 5 minutes.

By continuing the survey, you agree to the following statements:

1. I understand that my answers will not be disclosed to anyone and that my identity will remain anonymous. My name will not be written on the survey, nor will it be kept on any other records.
2. I declare that I am a student currently studying at a university in Arab-speaking country.
3. When the results of the study are reported, I will not be identified by name or by any other information that can be used to infer my identity. Researchers will only have access to view any data collected during this research, but the data cannot be linked to me.
4. I have been informed that the research adheres to all generally recognized ethical rules and that the research project has been reviewed and approved by the Applied Science University in Jordan
5. I have read and understood all statements contained in this form.
6. I voluntarily agree to participate in this research project by completing the following questionnaire.

**Informed Consent Item (Mandatory)**

**Do you agree to participate in this study?**

- **Yes**
- **No**

**TAME-ChatGPT scale as adopted from:**

Sallam M, Salim NA, Barakat M, Al-Mahzoum K, Al-Tammemi AB, Malaeb D, Hallit R, Hallit S. Assessing Health Students' Attitudes and Usage of ChatGPT in Jordan: Validation Study. JMIR Med Educ 2023;9:e48254; doi: 10.2196/48254; PMID: 37578934

**Usage scale:**

1. **Perceived usefulness**
2. ChatGPT helps me to save time when searching for information
3. For me, ChatGPT is a reliable source of accurate information
4. I recommend ChatGPT to my colleagues to facilitate their academic duties
5. ChatGPT is more useful than other sources of information that I have used previously
6. I appreciate the accuracy and reliability of the information provided by ChatGPT
7. I believe that using ChatGPT can save time and effort in my university assignments and duties
8. **Behavior/cognitive factors**
9. I have used tools or techniques similar to ChatGPT in the past
10. I spontaneously find myself using ChatGPT when I need information for my university assignments and duties
11. I often use ChatGPT as a source of information in my university assignments and duties
12. **Perceived risk of use** REVERSED SCORE
13. I am concerned that using ChatGPT would get me accused of plagiarism (REVERSED SCORE)
14. I am concerned about the potential security risks of using ChatGPT (REVERSED SCORE)
15. I think that relying on technology like ChatGPT can disrupt my critical thinking skills (REVERSED SCORE)
16. **Perceived ease of use**
17. It does not take a long time to learn how to use ChatGPT
18. ChatGPT does not require extensive technical knowledge

**Heard of ChatGPT but did not use it**

1. **Perceived risk** REVERSED SCORE
2. I am concerned about the reliability of the information provided by ChatGPT (REVERSED SCORE)
3. I am concerned that using ChatGPT would get me accused of plagiarism (REVERSED SCORE)
4. I am concerned about the potential security risks of using ChatGPT (REVERSED SCORE)
5. I am afraid that the use of the ChatGPT would be a violation of academic and university policies (REVERSED SCORE)
6. I am concerned about the potential privacy risks that might be associated with using ChatGPT (REVERSED SCORE)
7. **Anxiety** REVERSED SCORE
8. I am afraid of relying too much on ChatGPT and not developing my critical thinking skills (REVERSED SCORE)
9. I am afraid of becoming too dependent on technology like ChatGPT (REVERSED SCORE)
10. I am afraid that using ChatGPT would result in a lack of originality in my university assignments and duties (REVERSED SCORE)
11. **Technology/social influence**
12. I am enthusiastic about using technology such as ChatGPT for learning and research
13. I believe technology such as ChatGPT is an important tool for academic success
14. I think that technology like ChatGPT is attractive and fun to use
15. I am always keen to learn about new technologies like ChatGPT
16. I trust the opinions of my friends or colleagues about using ChatGPT

**Note 1.** Each question is scored on a 5-point Likert scale (agree, somewhat agree, neutral/no opinion, somewhat disagree, disagree)

**Note 2.** Two items are repeated in the Usage and Hearing scales
